# Retraction Note: May i come in? a probe into the contributions of self-esteem, teacher support, and critical thinking to anxiety and shyness in language classes

**DOI:** 10.1186/s40359-025-02866-y

**Published:** 2025-05-20

**Authors:** Lei Li, Tahereh Heydarnejad

**Affiliations:** 1 School of History and Culture, Northeast Normal University, Changchun, 130024 People’s Republic of China; 2 Department of English Language, Faculty of Literature and Humanities, University of Gonabad, Gonabad, Iran

**Retraction Note: BMC Psychology (2023) 12:7**

10.1186/s40359-023-01501-y

The editor and the publisher have retracted this article. The article was submitted to be part of a guest-edited issue. An investigation by the publisher found a number of articles, including this one, with a number of concerns, including but not limited to compromised editorial handling and peer review process, inappropriate or irrelevant references or not being in scope of the journal or guest-edited issue. Based on the investigation’s findings the editor therefore no longer has confidence in the results and conclusions of this article.

Author Tahereh Heydarnejad disagrees with this retraction. Author Lei Li has not responded to correspondence regarding this retraction.

